# SFRP4 promotes autophagy and blunts FSH responsiveness through inhibition of AKT signaling in ovarian granulosa cells

**DOI:** 10.1186/s12964-024-01736-1

**Published:** 2024-08-14

**Authors:** Michael Bérubé, Atefeh Abedini, Evelyne Lapointe, Samuel Gusscott, Julie Brind’Amour, Gustavo Zamberlam, Derek Boerboom

**Affiliations:** Centre de Recherche en Reproduction et Fertilité, Faculté de Médecine Vétérinaire, Université de Montréal, 3200 rue Sicotte, St-Hyacinthe, QC J2S 2M2 Canada

**Keywords:** SFRP4, Granulosa cells, Gonadotropins, Autophagy, KO mice, Ovary, RNA-Seq

## Abstract

**Background:**

Secreted frizzled-related proteins (SFRPs) comprise a family of WNT signaling antagonists whose roles in the ovary are poorly understood. *Sfrp4*-null mice were previously found to be hyperfertile due to an enhanced granulosa cell response to gonadotropins, leading to decreased antral follicle atresia and enhanced ovulation rates. The present study aimed to elucidate the mechanisms whereby SFRP4 antagonizes FSH action.

**Methods:**

Primary cultures of granulosa cells from wild-type mice were treated with FSH and/or SFRP4, and effects of treatment on gene expression were evaluated by RT-qPCR and RNAseq. Bioinformatic analyses were conducted to analyse the effects of SFRP4 on the transcriptome, and compare them to those of FSH or a constitutively active mutant of FOXO1. Additional granulosa cell cultures from wild-type or *Sfrp4*-null mice, some pretreated with pharmacologic inhibitors of specific signaling effectors, were used to examine the effects of FSH and/or SFRP4 on signaling pathways, autophagy and apoptosis by western blotting and TUNEL.

**Results:**

Treatment of cultured granulosa cells with recombinant SFRP4 was found to decrease basal and FSH-stimulated mRNA levels of FSH target genes. Unexpectedly, this effect was found to occur neither via a canonical (CTNNB1-dependent) nor non-canonical WNT signaling mechanism, but was found to be GSK3β-dependent. Rather, SFRP4 was found to antognize AKT activity via a mechanism involving AMPK. This lead to the hypophosphorylation of FOXO1 and a decrease in the expression of a portion of the FSH and FOXO1 transcriptomes. Conversely, FSH-stimulated AMPK, AKT and FOXO1 phosphorylation levels were found to be increased in the granulosa cells of *Sfrp4*-null mice relative to wild-type controls. SFRP4 treatement of granulosa cells also induced autophagy by signaling via AKT-mTORC1-ULK1, as well as apoptosis.

**Conclusions:**

This study identifies a novel GSK3β-AMPK-AKT signaling mechanism through which SFPR4 antagonizes FSH action, and further identifies SFRP4 as a novel regulator of granulosa cell autophagy. These findings provide a mechanistic basis for the phenotypic changes previously observed in *Sfrp4*-null mice, and broaden our understanding of the physiological roles of WNT signaling processes in the ovary.

**Supplementary Information:**

The online version contains supplementary material available at 10.1186/s12964-024-01736-1.

## Background

Autophagy is an evolutionarily conserved catabolic process that ensures the degradation and recycling of cellular components that are dysfunctional or no longer needed. Originally described as an adaptive response to nutrient deprivation, it is now clear that autophagy plays key roles in many physiological processes, including cell proliferation, differentiation and apoptosis [1–3]. Ovarian follicle development is a process that relies heavily on autophagy at multiple stages, and it occurs in both the oocyte and the somatic cells [4, 5]. For instance, autophagy is involved in the breakdown of germ cell cysts and the formation of primordial follicles in the perinatal mouse ovary [6, 7]. Granulosa cell differentiation and estradiol biosynthesis have recently been shown to require the autophagy-mediated degradation of the transcription factor WT1 [8]. Follicular atresia also requires autophagy, and recent evidence suggests that complex interactions exist between autophagy and apoptosis pathways in granulosa cells during this process [9, 10]. In addition, autophagy participates in both luteinization and luteal regression [11–14].

Although autophagy can be induced via multiple pathways, PI3K/AKT/mTOR appears to play a central role during ovarian follicle development [15]. In response to stimuli including nutrients, growth factors and hypoxia, mTORC1 can phosphorylate and inactivate the ULK complex, thereby inhibiting autophagy [16]. AMPK can also regulate this process, either by phosphorylating (and thereby inactivating) mTORC1, or through a direct activating phosphorylation of ULK1 [16–19]. Upon activation, the ULK complex phosphorylates BECN1, itself part of a complex that includes the class III PI3 kinase Vps34. Upon relocalization of these complexes to the phagophore, PI3P synthesis by the BECN1-PI3K complexes allows for the docking of additional proteins, phagophore maturation and the formation of autophagosomes [20]. At this time, LC3-I is converted to LC3-II by conjugation to phosphatidylethanolamine and recruited to autophagosomal membranes, a step which is considered a hallmark of autophagosome formation. LC3 then functions together with SQSTM1 in the capture of cargo for autophagic degradation [21, 22]. Autophagosomes subsequently fuse with lysosomes, resulting in the degradation of intra-autophagosomal components. In the ovary, FSH has been shown to activate AKT in follicular granulosa cells, which in turn phosphorylates mTOR to suppress autophagy [23, 24], thereby inhibiting apoptosis/atresia. This effect may however be context- or dose-dependent, as additional studies have shown the induction of autophagy by FSH [25, 26]. LH has also been shown to induce mTOR (and suppress AMPK) activity to suppress autophagy in luteal cells [12]. Unlike FSH however, LH seems to exert these effects through modulation of GSK3β and AMPK activity, rather than PI3K/AKT [27].

Despite the importance of FSH in ovarian physiology, its post-receptor mechanisms of action remain incompletely understood. Following FSH binding to its receptor, a number of intracellular signaling cascades are activated, most notably the PKA and PI3K/AKT pathways. In the PKA pathway, activation of adenylyl cyclase activity results in increased intracellular cAMP levels, leading in turn to the activation of PKA. The best-characterized substrate of the latter is cAMP response element-binding protein (CREB), a transcription factor which, upon phosphorylation and translocation to the nucleus, binds cAMP response elements present in the promoters of genes such as *Cyp19a1* and *Star* to increase their transcriptional activity [28, 29]. Following activation by FSH, AKT acts to phosphorylate several signaling effectors, including the transcription factor forkhead box protein O1 (FOXO1). Phosphorylation promotes the export of FOXO1 from the nucleus, thereby relieving its inhibition of transcription of genes including *Ccnd2, Cyp19a1*, *Lhcgr* and *Nppc* [30, 31]. Importantly, FOXO1 has also recently emerged as an important regulator of autophagy, acting not only in the nucleus to regulate the expression of several autophagy-related genes, but also by associating in the cytosol with the autophagosome assembly factor ATG7 following its acetylation [32]. Indeed, FSH-mediated protection from autophagy following oxidative stress in granulosa cells seems to be mediated in large part by FOXO1, acting both via transcriptional and non-transcriptional mechanisms [23].

The canonical WNT signaling pathway has recently been identified as a key mediator of the follicular response to both FSH and LH [33–42]. In this pathway, the binding of WNT ligands to FZD-LRP5/6 receptor/co-receptor complexes results in the inactivation of GSK3β, allowing the transcriptional co-regulator CTNNB1 (β-catenin) to escape degradation. It can subsequently translocate to the nucleus where it interacts with various transcription factors to modulate the transcriptional activity of target genes [36, 43]. Importantly, aside from WNTs, both FSH and LH can also promote CTNNB1 stabilization in granulosa cells [33, 34, 37]. In the ovary, *Cyp19a1* and *Lhcgr* have both been shown to require CTNNB1 for optimal expression in response to FSH [37, 38]. Sustained overexpression of CTNNB1 in granulosa cells has also been shown to enhance FSH-stimulated follicle growth, increase FSH target gene expression, decrease follicular atresia and promote the development of granulosa cells tumors [39, 44]. Recently, we have identified the WNT signaling antagonist gene *Sfrp4* as a negative regulator of ovarian function, with *Sfrp4*-null mice notably being characterized by decreased follicular atresia, increases ovulation rates and female hyperfertility [45]. In addition, the expression of FSH-responsive genes was found to be increased in the preovulatory follicles of *Sfrp4*-null mice, as was the expression of LH-responsive genes in ovulating follicles, suggesting that *Sfrp4* acts as an inhibitor of gonadotropin action [33, 45]. However, the mechanism of SFRP4 action in this context remains to be defined. *SFRP4* is also expressed in the ovary in women, although its expression pattern is different from that observed in mice. In the human ovary, *SFRP4* expression decreases as follicles grow to the preovulatory stage, with higher expression occurring in cumulus GCs (suggesting a role in oocyte maturation), whereas in mice follicular *Sfrp4* expression is induced by gonadotropins [45, 46]. SFRP4 has also been detected in plasma in women at a concentration of approximately 4 ng/mL [47].

In the present article, we report that SFRP4 antagonizes FSH activity not through the expected CTNNB1-mediated mechanism, but rather though suppression of AKT/FOXO1, and also define it as a novel regulator of autophagy in mouse ovarian granulosa cells.

## Materials and methods

### Ethics and animal models

All animal procedures were approved by the institutional animal care and use committee and conformed to the International Guiding Principles for Biomedical Research Involving Animals.

C57BL/6J wild-type (referred to herein as WT) mice were obtained from the Jackson Laboratory (Bar Harbor, ME, USA). *Sfrp4*-null mice were generated and genotyping analyses done as previously described [45].

### Granulosa cell isolation and culture

All materials were obtained from Life Technologies Inc. (Burlington, ON, Canada) unless otherwise stated. GCs for primary culture were obtained from immature (21-26 day old) WT or *Sfrp4*-null mice 48 h after administration of equine chorionic gonadotropin (eCG, Folligon, Intervet, Kirkland, Québec, Canada, 5IU, IP). Granulosa cells (GCs) were isolated by placing the ovaries in MEM 1X and puncturing the follicles with 26-gauge needles. GCs were pooled, followed by filtration using 40μm cell strainers (Progene 71-229481-ULT, Ultident, St-Laurent, QC, Canada) to remove COCs, and centrifugation for 10 min at 2000g. The cell pellet obtained was resuspended in MEM containing 1% fetal bovine serum, 0.25 nM sodium pyruvate, and 3 mM L-glutamate. GCs were seeded in 96-well culture plates (each well receiving the granulosa cells isolated from 0.5 ovary) and incubated for 3h at 37ºC in 5% CO_2_/95% air. Culture medium was then replaced with serum-free medium for 2h, and the GCs treated (or not) with exogenous SFRP4 (20 ug/mL; #1827-SF-025; R and D Systems; Bio-Techne; Minneapolis; MN) for 1 hour [except for the time- and dose response experiments, in which SFRP4 concentrations (10 ng to 20 ug) and treatment durations (15 minutes to 4 hours) varied], prior to addition (or not) of recombinant human FSH (50 ng/mL; National Hormone and Peptide Program, Torrance, CA) for 1h (western blotting experiments) or 2h (RT-qPCR experiments). For some experiments, small molecule inhibitors (Tocris Bioscience, Bristol, United Kingdom) were added to the culture medium 1h prior to addition of SFRP4. These included SP600125 (#1496, 100 μM), GF109203X (#0741, 30 μM), KN-93 (#1278, 2.5 μM), Xestospongin C (#1280, 5 μM) and SB216763 (#1616, 1 μM). Either immediately after isolation or following culture, GCs were lysed with RLT buffer (Qiagen, Hilden, Germany) or SDS loading buffer containing 5% β-Mercaptoethanol (BioShop, Burlington, ON) and stored at -80ºC prior to RNA extraction or immunoblotting.

### Real-time RT-PCR

Total RNA from granulosa cells was extracted using the RNeasy Mini Kit (Qiagen) according to the manufacturer’s protocol. Reverse transcription was done using 200 ng of RNA and the SuperScript^TM^ IV Vilo^TM^ cDNA synthesis kit (#11756500, Invitrogen). Real-time PCR was done using CFX96 Touch™ instrument (Bio-Rad). Each PCR reaction consisted of 7.5 μl of Advanced SYBR Green PCR Master Mix (#800-435 QL; Wisent, QC, CA), 2.3 μl of water, 4 μl of cDNA sample (diluted 10-fold from the RT reactions), and 0.6 μl (60 pmol) of gene-specific primers (listed in Supplemental Table 1). Cycling conditions were the same in all cases and as previously described [45]. To quantify relative gene expression, the Ct of target gene amplification was normalized to the expression level of a housekeeping gene (*Rpl19*) according to the ratio, *R*= E^Ct Rpl19^/E^Ct target^ [48], where E is the amplification efficiency for each primer pair.

### Immunoblotting

Samples underwent electrophoresis on 4-20% precast polyacrylamide gels (#5678095; Biorad; Hercules, CA) and were subsequently transferred to Low-autofluorescence PVDF membranes (#1704275; Biorad). After transfer, the membranes were blocked using 5% non-fat dry milk in Tris-buffered saline with 0.1% Tween 20 (#1706531; BioShop) (TBST) for 1h before being probed with antibodies raised against phosphorylated AKT (#9271), total AKT (#9272), phosphorylated AMPKα (#2535), total AMPKα (#2532), phosphorylated BECN1 (#35955), total BECN1 (#3495), phosphorylated CTNNB1 (Ser33/37/Thr41) (#PAS-17915, Thermofisher, Waltham, MA), non-phosphorylated (Ser33/37/Thr41) (i.e., active) CTNNB1 (#8814), phosphorylated CTNNB1 (Ser552) (#9566), total CTNNB1 (#51967-2-AP; 1:50000, ProteinTech, Rosemont, IL), Cleaved Caspase-3 (#9661), phosphorylated CREB (#9198), total CREB (#9197), phosphorylated FOXO1 (#9461), total FOXO1 (#2880), phosphorylated GSK3β (#5558), total GSK3β (#9315), LC3B (#3868), phosphorylated mTOR (#2971), total mTOR (#2983), SQSTM1/p62 (#23214), phosphorylated ULK1 (ser555) (#5869) and (ser757) (#6888), total ULK1 (#8054) or ACTB (#sc-47778; 1:10000; Santa Cruz Biotechnology, Dallas, TX) diluted in 5% bovine serum albumin. All antibodies mentioned above were obtained from Cell Signaling Technology (Danvers, MA) and used at a 1:1000 dilution unless otherwise specified. Membranes were incubated with primary antibody overnight. After washing three times with TBST, membranes were incubated for 1 h at room temperature with anti-rabbit HRP-conjugated IgG (GE Healthcare Life Sciences; Baie d’Urfé, Canada) diluted 1:10000 in 5% non-fat dry milk in TBST. In some instances, membranes were sequentially stripped with Restore Western Blot Stripping Buffer (#21059; Thermo Scientific; Waltham, MA) and re-probed with other antibodies. Protein bands were visualized by chemiluminescence (ECL; Millipore Sigma; Darmstadt, Germany) and quantified using a ChemiDoc MP detection system (Bio-Rad) and Image Lab™ software.

### TUNEL assays

GCs from 48h eCG-primed mice were pretreated (or not) with SFRP4 recombinant protein (20 ug/mL) for 1h and/or FSH (50 ng/mL) for 4 hours in 8 well chamber slides (4 wells/treatment group). After the treatments, cells were fixed in 4% paraformaldehyde for 1 hour. Subsequent steps of permeabilization and labeling were conducted using the In Situ Cell Death Detection Kit, TMR red, following the manufacturer's instructions (Roche Molecular Biochemicals, Millipore Sigma). Slides were mounted using VectaShield with 4′,6′-diamidino-2-phenylindole (Vector Laboratories, Burlingame, CA). The percentage of dead cells was quantified using ImageJ 1.53a [49] software as previously described [50] on monochrome images (*n*=20/treatment).

### RNA-seq library preparation

Briefly, total RNA was isolated from cultured granulosa cells from WT mice that had been treated (or not) with recombinant SFRP4 (20 μg/ml) for 3h, as described above, in biological triplicates. 250 ng RNA was used for ribosomal RNA depletion using the NEBNext rRNA Depletion Kit (#E6310X) according to the manufacturer’s instructions. Double-stranded cDNA was then synthesized using the NEBNext Ultra II RNA First Strand Synthesis (#E7771) and the NEBNext Ultra II Directional RNA Second Strand Synthesis (#E7550L) modules. Libraries were constructed using NEBNext Ultra II End Repair/dA-Tailing (#E7546L) and NEBNext Ultra II Ligation (#E7595L) modules. Each library was amplified for 9 PCR cycles using NEBNext High-Fidelity 2X PCR Master Mix (#M0541L) and NEBNext Multiplex Oligos for Illumina (UDI) (#E6440S). Libraries were pooled and 150 bp paired-end sequencing was carried out on the NovaSeq 6000 platform at The Center for Applied Genomics (Toronto, Ontario, Canada) according to the manufacturer’s instructions.

### RNA-seq data processing and analysis

Fastq data was processed using the GenPipes v.3.1.2 pipeline [51]. Briefly, raw reads were quality and adaptor trimmed using Trimmomatic, then aligned to the mm10 (mouse) reference genome using the STAR aligner. Library technical quality was assessed using Picard-tools (http://broadinstitute.github.io/picard) v.1.92 and Samtools (http://www.htslib.org/) v.1.1. PCR duplicates and fragments with poor mapping quality (q < 255) were filtered out. Gene transcription levels were quantitated over annotated Ensembl genes, and differential gene expression was measured between the control and SFRP4 condition using DESeq2 (corrected *p*-value < 0.05, LOG_2_ fold-change > 2). Differentially expressed genes (DEGs) underwent Gene Set Enrichment Analysis using DAVID (https://david.ncifcrf.gov/home.jsp) [52, 53] bioinformatics tool to identify biological processes and KEGG pathways related to these genes. Selected biological processes and pathways were regrouped in band graphics and bubble plots using SRplot (https://www.bioinformatics.com.cn/srplot) [54]. Full lists of gene ontology (GO) biological processes and pathways are provided in supplemental Tables 2 to 5. The SFRP4 up- and downregulated gene groups were also compared with previously-described data sets generated using granulosa cells treated with FSH or expressing a constitutively stable and active mutant of FOXO1 [31]. Overlap between SFRP4, FSH and FOXO1 target genes was visualized using the Venny diagram online tool (https://csbg.cnb.csic.es/BioinfoGP/venny.html) [55]. Gene Set Enrichment Analysis of the overlapping DEGs was performed using Metascape (www.metascape.org) [56].

### Statistical analyses

All statistical analyses were performed using Prism 4.0a (GraphPad Software Inc.; La Jolla, CA) software. All the data sets (mRNA expression and protein expression) were subjected to the F-test to determine equality of variances. Data were transformed to logarithms if they were not normally distributed. Two-tailed t-tests were used when two experimental groups were compared, or ANOVA (with Tukey’s multiple comparisons post-test) to compare three or more groups. All data are presented as means ± SEM.

## Results

### SFRP4 blunts granulosa cell responsiveness to FSH* in vitro*

**Fig. 1:**
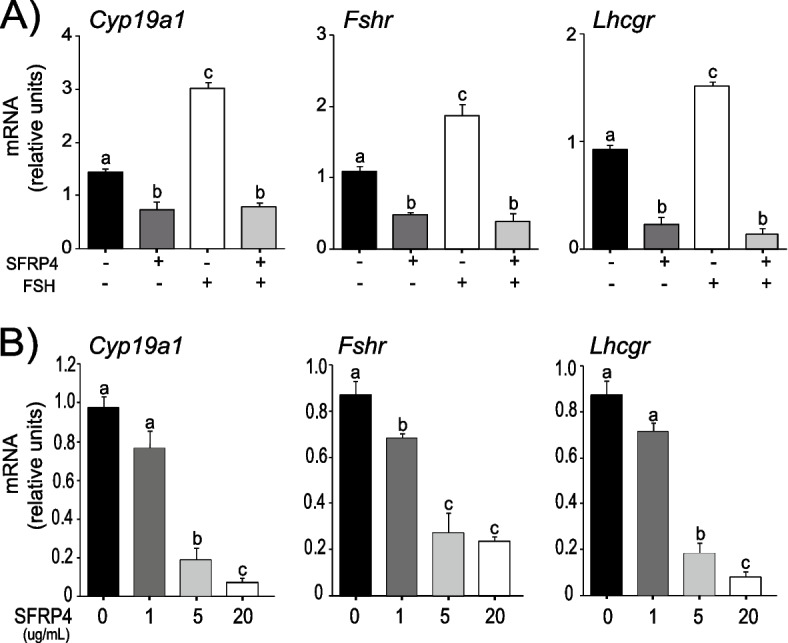
SFRP4 regulates FSH-responsive genes in GCs in a dose-dependent manner. **A** GCs were isolated from immature (21-26 days-old) eCG-primed wild-type mice and placed in culture. GCs were pre-treated for 1h with recombinant SFRP4 and then challenged with FSH for 2h. **B** eCG-primed immature wild-type granulosa cells were treated with graded doses of recombinant SFRP4 up to 20 ug/mL. mRNA levels for each FSH target gene were determined by RT-qPCR and normalized to the housekeeping gene *Rpl19* (*n*=3-4 samples/group). Data are expressed as means ± SEM. Groups not labeled with a common letter were significantly different (*P <* 0.05)

To elucidate the mechanisms whereby *Sfrp4* inhibits follicle survival and gonadotropin responsiveness, a granulosa cell primary culture system was first developed to recapitulate the effects of *Sfrp4* previously observed *in vivo* [45]. Granulosa cells isolated from eCG-primed immature mice were placed in culture and treated with recombinant SFRP4, followed or not by FSH treatment. SFRP4 treatment was found to reduce the basal mRNA levels of the FSH target genes *Cyp19a1*, *Fshr* and *Lhcgr*, and completely counteracted the ability of FSH to induce their expression (Fig. 1a). The effect of SFRP4 on the basal mRNA levels of these genes was found to be dose-dependent (Fig. 1b).

### The mechanism of action of SFPR4 is GSK3β-dependent, but independent of CTNNB1

**Fig. 2:**
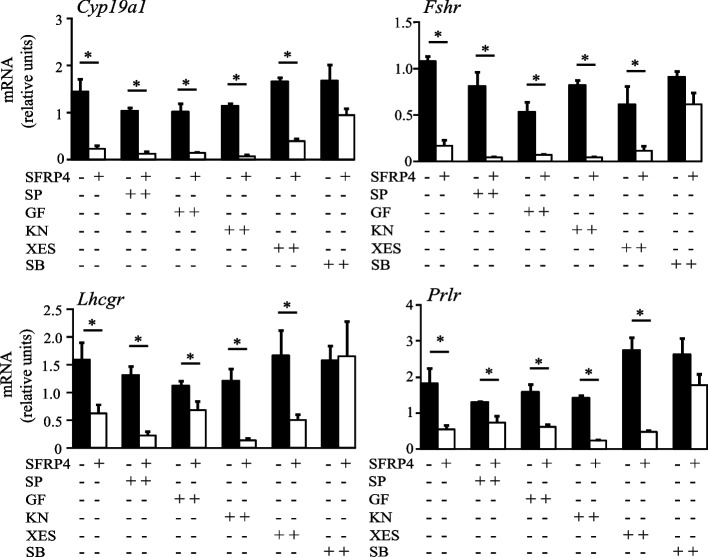
SFRP4 regulation of its target genes is GSK3β-dependent. GCs were isolated from immature (21-26 days-old) eCG-primed wild-type mice and placed in culture with recombinant SFRP4 protein (20 μg/ml) for 3h, with or without pretreatment with different pharmacologic inhibitors of WNT signaling effectors: SP (SP600125; JNK inhibitor), GF (109203X; PKC inhibitor), KN (KN93; CAMKII inhibitor), XES (xestospongin C; IP3-dependent calcium release inhibitor); or SB (SB216763; GSK3β inhibitor) for 1h. mRNA levels for each gene were determined by RT-qPCR and normalized to the housekeeping gene *Rpl19* (*n*=3-4 samples/treatment). Data are expressed as means ± SEM. Asterisks indicate statistically significant differences between groups (*P <* 0.05)

To identify the signaling pathway(s) downstream of SFRP4 responsible for its antagonism of FSH response gene expression, granulosa cells were pretreated with pharmacologic inhibitors of different WNT signaling pathways prior to SFRP4 treatment. Compounds targeting key components of the WNT/Ca^2+^ [KN-93 (KN, CAMKII inhibitor), Xestospongin C (XES, Ca^2+^ release inhibitor), GF109203X (GF, PKC inhibitor)] and planar cell polarity [SP600125 (SP, JNK inhibitor)] pathways all failed to counteract SFRP4-mediated decreases in FSH target gene mRNA levels. However, the GSK3β inhibitor SB216763 (SB) prevented the effects of SFRP4, suggesting that it acts via the canonical (i.e., WNT-CTNNB1) pathway (Fig. 2).

**Fig. 3:**
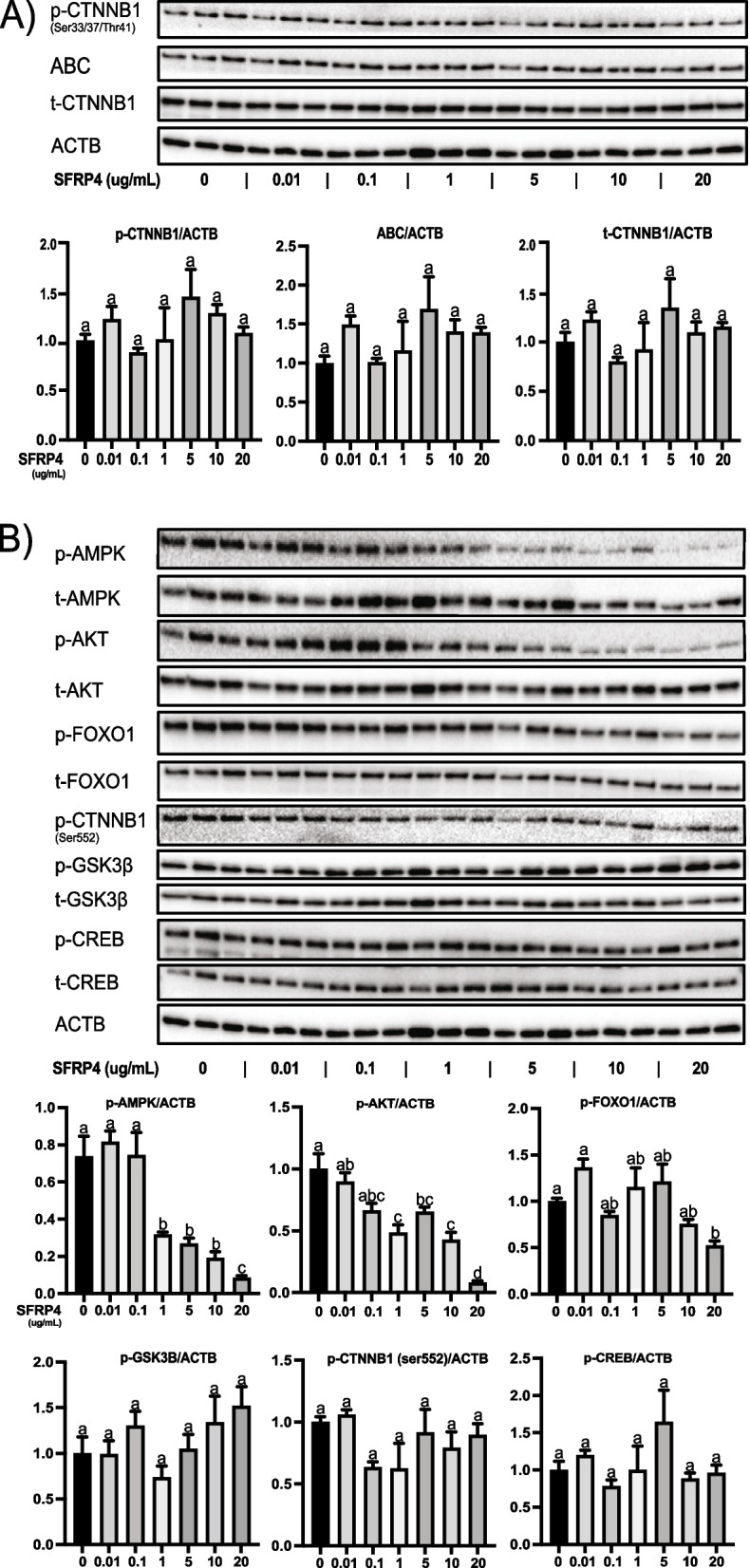
SFRP4 suppresses the activity of the AMPK-AKT axis but does not suppress CTNNB1 signaling *in vitro*. GCs were isolated from immature (21-26 days-old) eCG-primed wild-type mice, placed in culture without or with recombinant SFRP4 protein (up to 20 μg/ml) for 1h (*n* = 3 samples/treatment). Representative immunoblots of the indicated proteins and phosphoproteins are shown, along with densitometric analyses of the indicated phosphoproteins from the immunoblots (graphs). β-actin (ACTB) was used as the loading control. Data are expressed as means ± SEM. Groups not labeled with a common letter were significantly different (*P <* 0.05)

To confirm that SFRP4 signals via CTNNB1, granulosa cells were treated with graded doses of SFRP4, and protein levels of total CTNNB1, as well as CTNNB1 phosphorylated at its N-terminal tail by GSK3β (p-CTNNB1) and unphosphorylated CTNNB1 (“active CTNNB1”, ABC) were determined by immunoblotting. Unexpectedly, the expression of all forms of CTNNB1 was unaltered by even the highest doses of SFRP4, indicating that effector(s) downstream of GSK3β other than CTNNB1 are responsible for exerting its effects (Fig. 3a).

### SFRP4 acts via AMPK-AKT-FOXO1

**Fig. 4:**
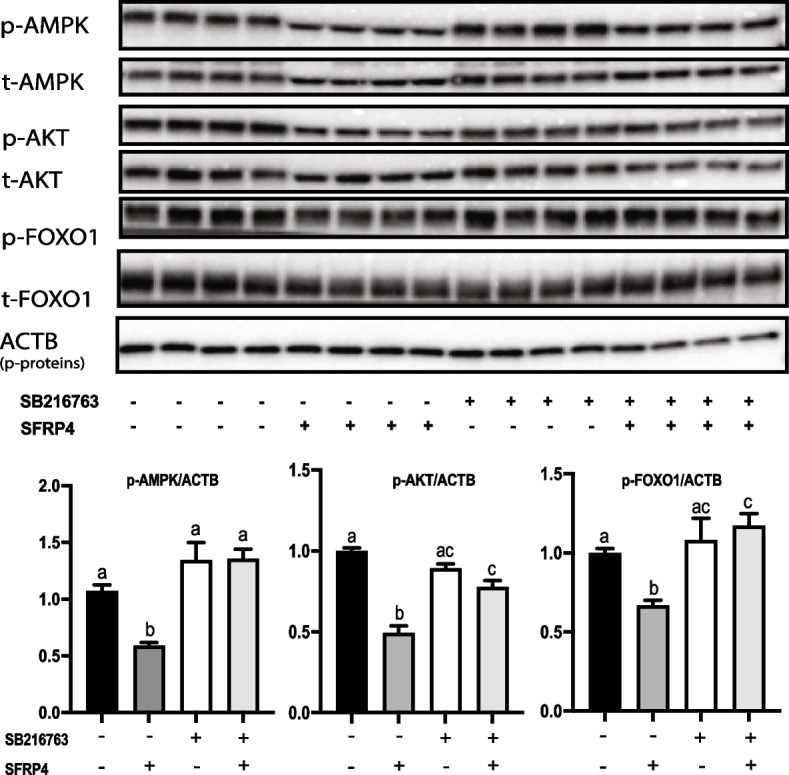
SFRP4-mediated downregulation of AMPK, AKT and FOXO1 is GSK3β-dependent. GCs isolated from immature (21-26 days-old) eCG-primed wild-type mice were pretreated with the GSK3β inhibitor SB216763 for 1h prior to treatment with recombinant SFRP4 for 2h (*n* = 4 samples/treatment). Representative immunoblots of the indicated proteins and phosphoproteins are shown, along with densitometric analyses of the indicated phosphoproteins from the immunoblots (graphs). β-actin (ACTB) was used as the loading control. Data are expressed as means ± SEM. Groups not labeled with a common letter were significantly different (*P <* 0.05)

To identify SFRP4 signaling mechanisms downstream of GSK3β, the expression of a number of signaling effectors known to be regulated by GSK3β were studied using the same dose-response assay described in Fig. 3a. These experiments identified AMPK as a downstream target of SFRP4, as phosphorylation of AMPK at Thr172 (required for its activation) was significantly decreased by SFRP4 concentrations as low as 1μg/ml (Fig. 3b). This effect was then shown to be GSK3β-dependent, as pretreatment of granulosa cells with SB216763 prevented SFRP4 from altering p-AMPK expression (Fig 4.). As AKT is both a key effector downstream of AMPK in the context of the cell stress response and a key effector of FSH signaling, pAKT expression in response to SFRP4 was assayed, and found to decrease at the same dose of SFRP4 as that which decreased pAMPK. Likewise, phosphorylation of the AKT substrate FOXO1 (a key transcriptional regulator of FSH target genes and a mediator of autophagy) decreased in response to SFRP4 (Fig. 3b). The effects of SFRP4 on both pAKT and pFOXO1 were sensitive to SB216763 (Fig. 4), indicating that both occur downstream of GSK3β. Unlike pAKT, the expression of the FSH signaling effector pCREB was not affected by SFRP4 treatment, nor was the phosphorylation of the AKT substrates GSK3β or CTNNB1 (at S552) (Fig. 3b).

**Fig. 5:**
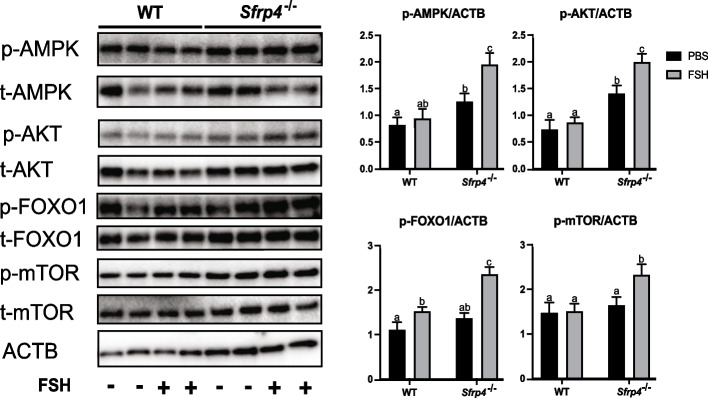
Levels of pAKT, pFOXO1, pAMPK and pmTOR are increased in GCs from *Sfrp4*-null mice. GCs isolated from immature (21-26 days-old) eCG-primed *Sfrp4*-null and wild-type mice were placed in culture and treated (or not) with FSH (50 ng/ml) for 1h (*n* = 4 samples/treatment). Immunoblots show results for the indicated proteins and phosphoproteins (*n*=2 samples/group), densitometric analyses of the indicated phosphoproteins from the immunoblots (graphs) include all four samples/group. β-actin (ACTB) was used as the loading control. Data are expressed as means ± SEM. Groups not labeled with a common letter were significantly different (*P <* 0.05)

To perform the converse experiment, granulosa cells isolated from either wild-type or *Sfrp4*-null littermate mice were placed in culture and treated or not with FSH. Immunoblotting analyses of these cells showed higher pAMPK, pAKT and pFOXO1 levels in FSH-stimulated cells from *Sfrp4*-null mice relative to controls, and higher basal levels also in the cases of pAMPK and pAKT (Fig. 5), indicating that *Sfrp4* normally acts to suppress this pathway. Together, these results define a novel GSK3β/AMPK/AKT/FOXO1 pathway through which SFRP4 may act to antagonize FSH signaling.

### SFRP4 induces autophagy and apoptosis

**Fig. 6:**
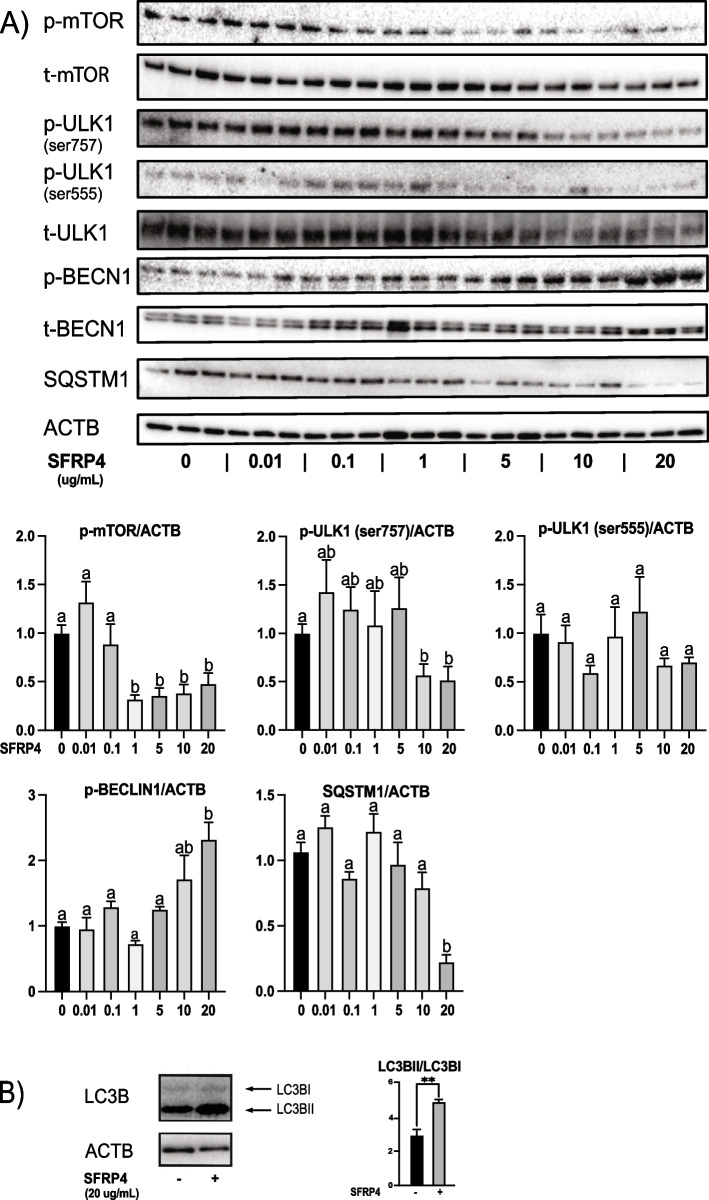
SFRP4 induces autophagy in GCs. GCs were isolated from immature (21-26 days-old) eCG-primed wild-type mice, and placed in culture without or with recombinant SFRP4 protein (up to 20 μg/ml) for 1h. A) Immunoblots show results for the indicated proteins and phosphoproteins (*n*=3 samples/group), along with densitometric analyses of the indicated phosphoproteins from the immunoblots (graphs). β-actin (ACTB) was used as the loading control. Groups not labeled with a common letter were significantly different (*P <* 0.05). B) Representative immunoblot of LC3B expression (*n*=1 sample/group), densitometric analyses (graph) quantify the ratio of signal strengths of the lower (LC3BII) to upper (LC3BI) bands, and include four samples/group for the quantification. Asterisks (**) indicate statistically significant differences between groups (*P <* 0.01). All data are expressed as means ± SEM

Another downstream effector of AKT is mTOR, the catalytic subunit of mTORC1. Its phosphorylation (and hence, activity) was also found to be suppressed by SFRP4 *in vitro* in a dose-dependent manner (Fig. 6a) and enhanced in granulosa cells from *Sfrp4*-null mice (Fig. 5), indicating that it is also a physiological effector of SFRP4 signaling. As mTORC1 is a key regulator of autophagy, autophagy is required for granulosa cell apoptosis and follicular atresia, and *Sfrp4*-null mice have reduced levels of follicular atresia, we tested the hypothesis that *Sfrp4* acts to regulate autophagy in granulosa cells.

Autophagy is normally suppressed by mTORC1 by phosphorylation of ULK1 at Ser757 [16]. Treatment of granulosa cells with SFRP4 *in vitro* resulted in dose-dependent suppression of ULK1 phosphorylation at Ser757, which is associated with the induction of autophagy (Fig. 6). Consistent with this, dose-dependent ULK1-mediated phosphorylation of BECN1 and degradation of SQSTM1 occurred in response to SFRP4 (Fig. 6a). Conversion of LC3B-I to LC3B-II was also observed following a 60 min treatment with SFRP4 (20 μg/mL) (Fig. 6b). AMPK can also activate ULK1 via direct phosphorylation at Ser555 [19], but (Ser555) pULK1 levels were not altered by SFRP4 treatment *in vitro* (Fig. 6a), suggesting that activation of autophagy by SFRP4 occurs via AMPK-mediated activation of AKT/mTOR, rather than its direct regulation of ULK1.

FOXO1 activity is regulated by SFRP4 (Figs. 3-5), and FOXO1 can regulate autophagy by transcriptional regulation of autophagy-related genes [32, 57–61]. We therefore determined the *in vitro* effect of SFRP4 on the mRNA levels of several key autophagy genes known to be regulated by FOXO1. These experiments failed to show SFRP4 to be able to induce the expression of any such genes, including *Atg3*, *Becn1*, *Map1lc3b* and *Ulk1* (Supp. Fig. 1). This result suggests that SFRP4 acts mostly by relieving the inhibition of ULK1 through mTOR to promote autophagy, rather than via transcriptional regulation of autophagy-related genes.

**Fig. 7:**
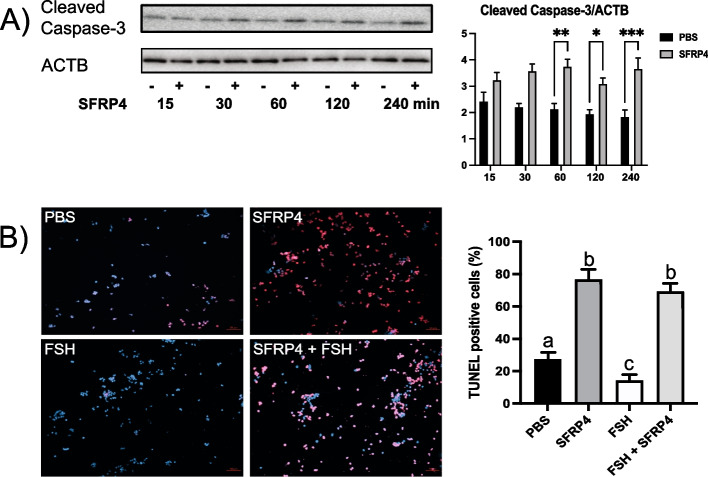
SFRP4 induces apoptosis in GCs *in vitro*. A) GCs were isolated from immature (21-26 days-old) eCG-primed wild-type mice, and placed in culture without or with recombinant SFRP4 on a time course (*n* = 4 samples/treatment). Representative immunoblot shows cleaved caspase-3 expression (*n*=1 sample/group/time), densitometric analyses (graph) include four samples/group. β-actin (ACTB) was used as the loading control. Asterisks indicate statistically significant differences between groups (*: *P <* 0.05, **: *P <* 0.01, ***: *P <* 0.001). Data are expressed as means ± SEM. B) GCs were isolated from immature (21-26 days-old) eCG-primed wild-type control mice, placed in culture with or without recombinant SFRP4 protein (20 μg/ml) for 1h, followed (or not) by FSH treatment (50 ng/ml, 4h, *n* = 4 samples/treatment). Photomicrographs show representative TUNEL analyses (red = TUNEL signal, blue = DAPI counterstain). Data are expressed as means ± SEM. Groups not labeled with a common letter were significantly different (*P <* 0.05)

Finally, to link the decrease in follicular atresia observed in *Sfrp4*-null mice to a potential pro-apoptotic action of *Sfrp4*, a timecourse analysis of cleaved caspase-3 expression was done using primary cultures of granulosa cells treated (or not) with SFRP4. Quantitative western blotting showed a significant increase in cleaved caspase-3 levels (i.e., apoptosis) starting at 60 minutes following addition of SFRP4 (Fig. 7A). Similar cultures of granulosa cells were treated (or not) with SFRP4 and/or FSH, and analyzed by TUNEL. As for the caspase-3 analyses, these assays showed that SFRP4 increased the proportion of cells undergoing apoptosis, either in presence or absence of FSH (Fig. 7B).

### Effects of SFRP4 treatment on the granulosa cell transcriptome

**Fig. 8:**
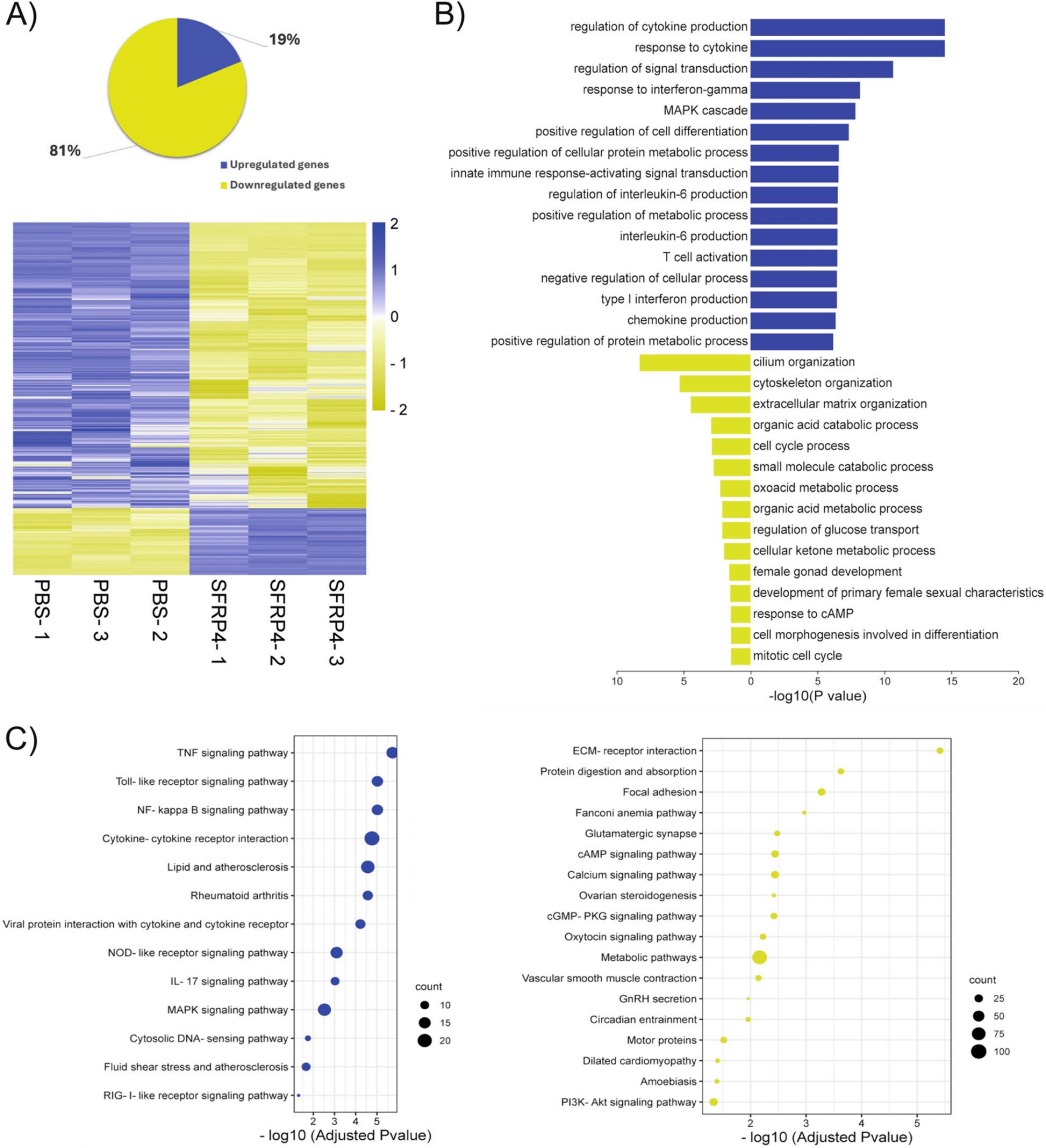
Exogenous SFRP4 regulates the expression of genes related to gonadotropin signaling and cell metabolism. **A** Differentially expressed genes (DEGs) identified by RNA-seq. The pie chart illustrates the proportion of up- and down-regulated genes among total DEGs induced by SFRP4 treatment. The heatmap displays the differential expression of all DEGs between control and treated groups. **B** Bar graphs depicting selected significantly enriched biological processes identified through David gene ontology analysis of up- and downregulated DEGs following SFRP4 treatment. **C** A bubble plot depicting selected significantly enriched pathways from David gene ontology analysis of up- and downregulated DEGs following SFRP4 treatment

To identify additional gene regulatory and biological processes impacted by SFRP4, an RNA-Seq analysis was performed using cultured granulosa cells from eCG-primed immature wild-type mice, treated or not with recombinant SFRP4. These analyses identified 2,897 transcripts whose expression was altered by SFRP4 treatment, including 463 transcripts with increased expression and 2,434 transcripts which decreased (log_2_ FC ≥ 2 and adjusted *P* value ≤ 0.05) (Figs S1, 8A). Gene ontology (GO) enrichment analysis of downregulated genes unveiled numerous FSH-relevant biological processes and pathways, including ovarian steroidogenesis, cAMP and PI3K-AKT signaling, cell division, and cellular metabolic activities. Conversely, upregulated genes showed associations with inflammation and protein metabolism (Fig. 8B, C). GO enrichment analysis also identified pathways pertinent notably to apoptosis and cytokine production, as well as additional autophagy-related genes known to be regulated by FOXO1 [60, 62, 63] (the complete lists of enriched biological processes and pathways are listed in Supp. Tables 2 to 5). However, RT-qPCR analyses failed to confirm the latter finding in most cases (Supp. Fig. 1), again suggesting that transcriptional regulation of autophagy-related genes is not a major means by which SFRP4 induces apoptosis in granulosa cells.

### Overlap between the SFRP4, FSH and FOXO1 transcriptomes

**Fig. 9:**
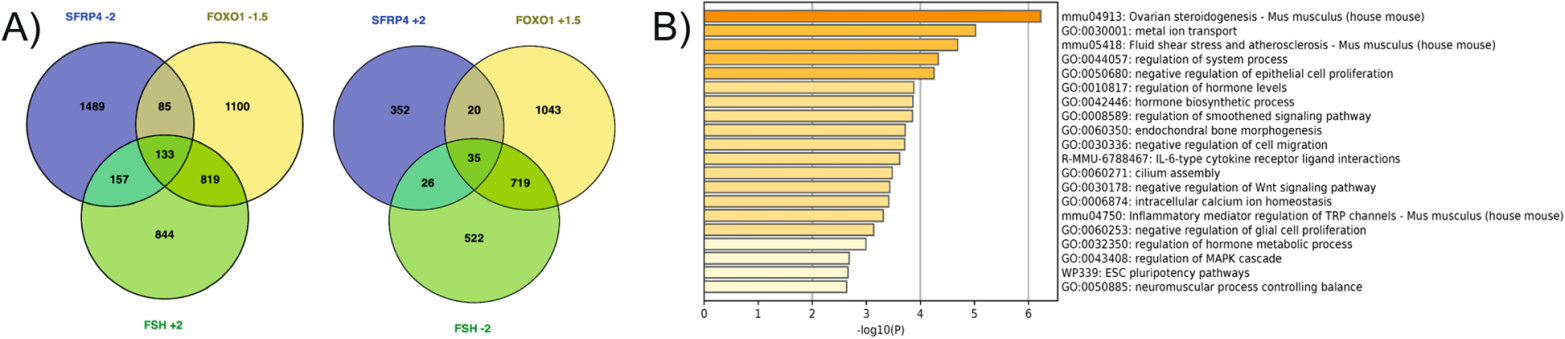
Overlap between DEGs following SFRP4 treatment vs DEGs following FSH treatment or expression of a constitutively active FOXO1 mutant in granulosa cells (GCs). **A** A Venn Diagram highlighting the overlap between DEGs induced by SFRP4 treatment and those induced by FSH treatment or FOXO1 in GCs. FSH and FOXO1 DEGs are derived from a previous study [31]. **B** A bar graph displaying the top 20 enriched Gene Ontology annotations for biological processes associated with common downregulated genes by SFRP4 or FOXO1 vs FSH upregulated genes. Several biological processes are related to ovarian steroidogenesis and follicular growth

To further explore the relationship between SFRP4, FSH and FOXO1, our SFRP4-treated granulosa cell RNA-seq data was compared to existing RNA-seq analyses of granulosa cells treated with FSH, or granulosa cells expressing a constitutively active mutant of FOXO1 [31]. These analyses identified a group of 133 genes commonly downregulated by SFRP4 and FOXO1 and upregulated by FSH (Fig. 9A, Supplemental table 6). A gene ontology enrichment analysis using Metascape related these genes to biological processes including ovarian steroidogenesis, cell proliferation and inflammation (Fig. 9B). Importantly, while overlap existed between the SFRP4, FSH and FOXO1 transcriptomes, the great majority of SFRP4-regulated genes were not regulated by FSH or FOXO1, and the extent of overlap between the SFRP4/FSH and SFRP4/FOXO1 transcriptomes was much smaller than the overlap between those of FSH and FOXO1 (Fig. 9A). Likewise, only a handful of genes were commonly upregulated by SFRP4 and FOXO1 and downregulated by FSH (Fig. 9A).

## Discussion

Several previous studies have identified WNTs and the canonical (CTNNB1) WNT signaling pathway as positive regulators of various facets of ovarian follicle development and function [34–43]. As SFRP4 is thought to antagonize WNT signaling notably by binding and sequesting WNT ligands in the extracellular space [64], it is perhaps unsurprising that *Sfrp4*-null mice were found to have enhanced follicle development and survival, ovulation rates and fertility [45]. This was accompanied by enhanced gonadotropin response gene expression in ovarian granulosa cells, suggesting a synergistic interaction between WNT and gonadotropin signaling mechanisms, although the nature of this interaction was not determined [36, 43, 65]. In the present study, we provide evidence that the mechanism of SFRP4 signaling in granulosa cells, while GSK3β-dependent, does not involve the regulation of CTNNB1 activity. This was unexpected given that several studies in other cell types have suggested a canonical signaling mechanism for SFRP4 [66–68], and that the only study published thus far that has examined SFRP4 signaling in granulosa cells has also suggested that it modulates CTNNB1 expression, albeit to increase it [69]. The latter finding differed from ours most likely due to the effects of SFRP4 on cultured granulosa being evaluated over several days, rather than the acute treatments used in the current study.

**Fig. 10:**
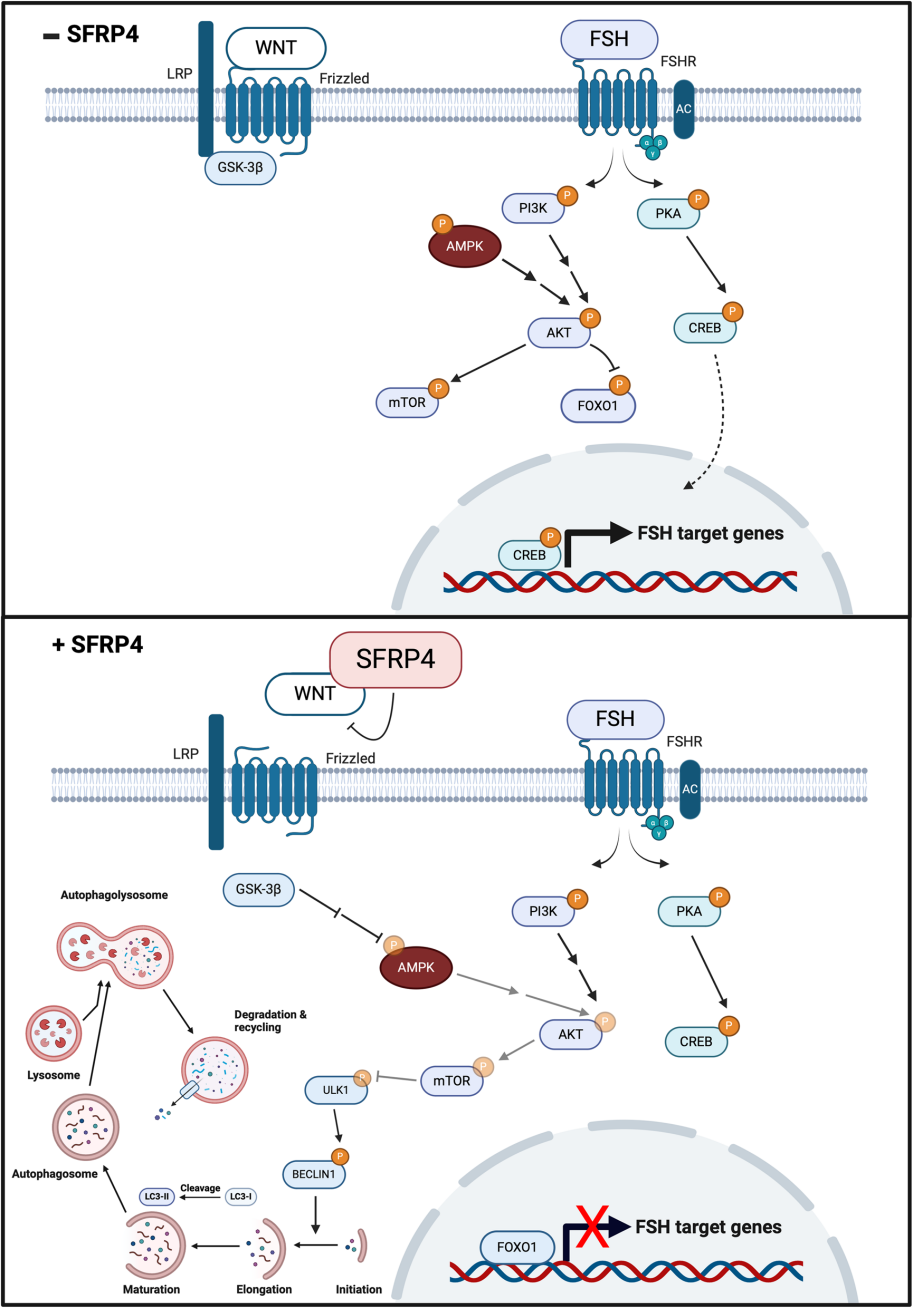
SFRP4 modulates FSH activity in granulosa cells by antagonizing the AMPK-AKT axis in a GSK3β-dependent manner. This antagonism leads to reduced AKT phosphorylation, resulting in decreased FOXO1 phosphorylation and enhanced nuclear accumulation. Concurrently, reduced AKT signaling downstream inhibits mTOR phosphorylation, thereby relieving inhibition of ULK1 and promoting autophagy. Pointed arrows indicate positive regulation, blunted arrows indicate negative regulation, double arrows indicate an indirect mechanism, pale arrows and phosphate groups (P) indicate decreases

Our subsequent experiments defined a GSK3β-AMPK-AKT-FOXO1 pathway through which SFRP4 appears to exert its effects both *in vitro* and *in vivo* (Fig. 10). Previous studies have shown FOXO1 to be a major effector of FSH-regulated gene expression in granulosa cells, and it has been proposed that FOXO1, together with CREB, regulate the expression of the great majority of FSH target genes [31]. Our RT-qPCR and RNA-seq analyses of SFRP4-treated granulosa cells did indeed reveal the regulation of a number of FSH- and FOXO1-regulated genes, as well as the regulation of FSH-relevant biological processes, suggesting that FOXO1 is a level at which SFRP4 acts to antagonize FSH signaling (Fig. 10). However, our data also indicate that SFRP4 can only modulate the expression of a relatively small fraction of the transcriptome previously reported to be regulated by FSH or by FOXO1 [31] in granulosa cells. Furthermore, the majority of SFRP4-regulated genes (and biological processes) identified in our RNA-seq analyses were not previously identified as being FSH- or FOXO1-regulated. Together, these findings suggest that SFRP4 1) may serve to antagonize only a subset of FSH/FOXO1 target genes and biological functions, 2) likely plays roles beyond antagonism of gonadotropin action, and 3) likely exerts its effects through a number of mechanisms in addition to the regulation of FOXO1 activity.

A key FSH-relevant biological processes that we found to be regulated by SFRP4 is granulosa cell autophagy. SFRP4-mediated induction of autophagy appears to occur through an AKT/mTOR/ULK1 pathway (Fig. 10). As FSH also uses this pathway to suppress autophagy [23, 24], this may represent a second level at which SFRP4 antagonizes FSH. Regulation of autophagy via WNT signaling has been widely reported in the scientific literature, and occurs in a variety of developmental and physiological contexts [70–72]. However, the vast majority of these findings involve canonical signaling mechanisms, such as the corepression of SQSTM1 by CTNNB1 [70, 73, 74]. Here, we show that SFRP4-mediated induction of autophagy does not appear to involve CTNNB1, but rather occurs via a signaling mechanism resembling a recently-elucidated GSK3β-AMPK pathway used by WNT3a to regulate autophagy in neurons [75]. Although our findings indicate that SFRP4 signals via FOXO1, and FOXO1 is a known regulator of autophagy-related genes in several cell types, we were unable to detect regulation of most autophagy-related genes by SFRP4 *in vitro*. While this suggests that this is not a major means by which SFRP4 induces autophagy, we cannot exclude that our experimental design was not optimal to detect this, for instance in terms of treatment times and culture conditions. Beyond regulation of the expression of autophagy-related genes, FOXO1 can also contribute to the induction of autophagy via its ability to interact with ATG7 [32, 59]. Whether or not this occurs specifically in granulosa cells in response SFRP4 remains to be determined.

In this report we also demonstrate the ability of SFRP4 to induce apoptosis in granulosa cells. As follicular atresia involves the death of granulosa cells by apoptosis, this observation provides a plausible explanation for the decrease in antral follicle atresia observed in *Sfrp4*-null mice, which in turn led to enhanced follicle development, increased ovulation rates and hyperfertility [45]. Some previous evidence has suggested a link between autophagy and granulosa cell apoptosis in the context of follicular atresia [9]. For instance, autophagy and apoptosis occur concomitantly in the granulosa cells of atretic follicles, and FSH, which inhibits follicular atresia, acts to suppress autophagosome formation [10]. However, other lines of evidence have suggested that granulosa cell death in the context of atresia can be caused by either apoptosis or by autophagy, and that the two processes are not necessarily linked. For instance, oxidative stress can cause granulosa cell death without the involvement of apoposis, but agents such as FSH and melatonin can rescue granulosa cells from oxidative stress-induced death by counteracting autophagy via PI3K-AKT-mTOR and FOXO1 [23, 76, 77]. It therefore remains to be determined whether or not SFRP4-induced autophagy and apoptosis are causally linked in granulosa cells. Nonetheless, the mechanism whereby SFRP4 induces apoptosis can presumably to be linked to its ability to antagonize FSH signaling, which is critical for follicle survival.

## Conclusions

In summary, this study provides novel insight into the mechanisms of SFRP4 action in ovarian granulosa cells. Its antagonism of FSH action was found to be related, at least in part, to its ability to block AKT signaling via an unexpected mechanism involving GSK3β and AMPK. This antagonism deprives FSH from its ability to regulate a subset of its target genes, as well as to suppress granulosa cell autophagy and apoptosis. Together, these findings not only provide a mechanistic basis for the phenotypic changes previously observed in *Sfrp4*-null mice, but also broaden our understanding of the physiological roles of WNT signaling processes in the ovary.

### Supplementary Information

Additional file 1 Supp.Figure 1. SFRP4 regulates FOXO1-dependent autophagy-related gene expression.

Additional file 2 Supp.Table 1. Oligonucleotide primer sequences.

Additional file 3 Supp.Tables 2-5. Complete lists of enriched biological processes and pathways.

Additional file 4 Supp.Table 6. Overlap between genes downregulated by SFRP4 and FOXO1 vs upregulated by FSH.

## Data Availability

The datasets generated and analysed during the current study are available in the GSE266715 repository [https://www.ncbi.nlm.nih.gov/gds].

## Abbreviations

AKT: Protein kinase B/Thymoma viral proto-oncogene 1
AMPK: AMP-activated protein kinase
ATG7: Autophagy related gene 7
BECN1: Beclin1
cAMP: Cyclic adenosine monophosphate
Ccnd2: Cyclin D2
CREB: CAMP-response element binding protein
CTNNB1: β-catenin
Cyp19a1: Cytochrome P450 family 19 subfamily A member 1
DEGs: Differentially expressed genes
eCG: Equine chorionic gonadotropin
FOXO1: Forkhead box O1
FZD: Frizzled
FSH: Follicle-stimulating hormone
GC: Granulosa cell
GSK3β: Glycogen synthase kinase 3 beta
KO: knock out
LC3: Microtubule associated protein 1 light chain 3
LH: Luteinizing hormone
Lhcgr: Luteinizing hormone/choriogonadotropin receptor
LRP: Lipoprotein receptor-related
mTORC1: Mammalian target of rapamycin complex 1
Nppc: Natriuretic peptide C
PI3K: Phosphatidylinositol 3-kinase
PI3P: Phosphatidylinositol-3-phosphate
PKA: Protein kinase A
Rpl19: Ribosomal protein L19
Ser: Serine
SFRP4: Secreted frizzled related protein 4
SQSTM1/p62: Sequestosome-1
Star: Steroidogenic acute regulatory protein
Thr: Threonin
ULK1: Unc-51 like autophagy activating kinase 1
VPS34: Vacuolar protein sorting 34/Class III Phosphatidylinositol 3-kinase
WNT: Wingless-type mouse mammary tumor virus integration site
WT: Wild type
WT1: Wilms’ tumor 1
