# Supplementary material for: SFRP4 promotes autophagy and blunts FSH responsiveness through inhibition of AKT signaling in ovarian granulosa cells
Source: Cell Commun Signal. 2024 Aug 14;22:396. doi: 10.1186/s12964-024-01736-1 (PMC11323480; doi:10.1186/s12964-024-01736-1)
Supplement: Supplementary file 1 — Additional file 1 Supp.Figure 1. SFRP4 regulates FOXO1-dependent autophagy-related gene expression. [file 12964_2024_1736_MOESM1_ESM.pdf]

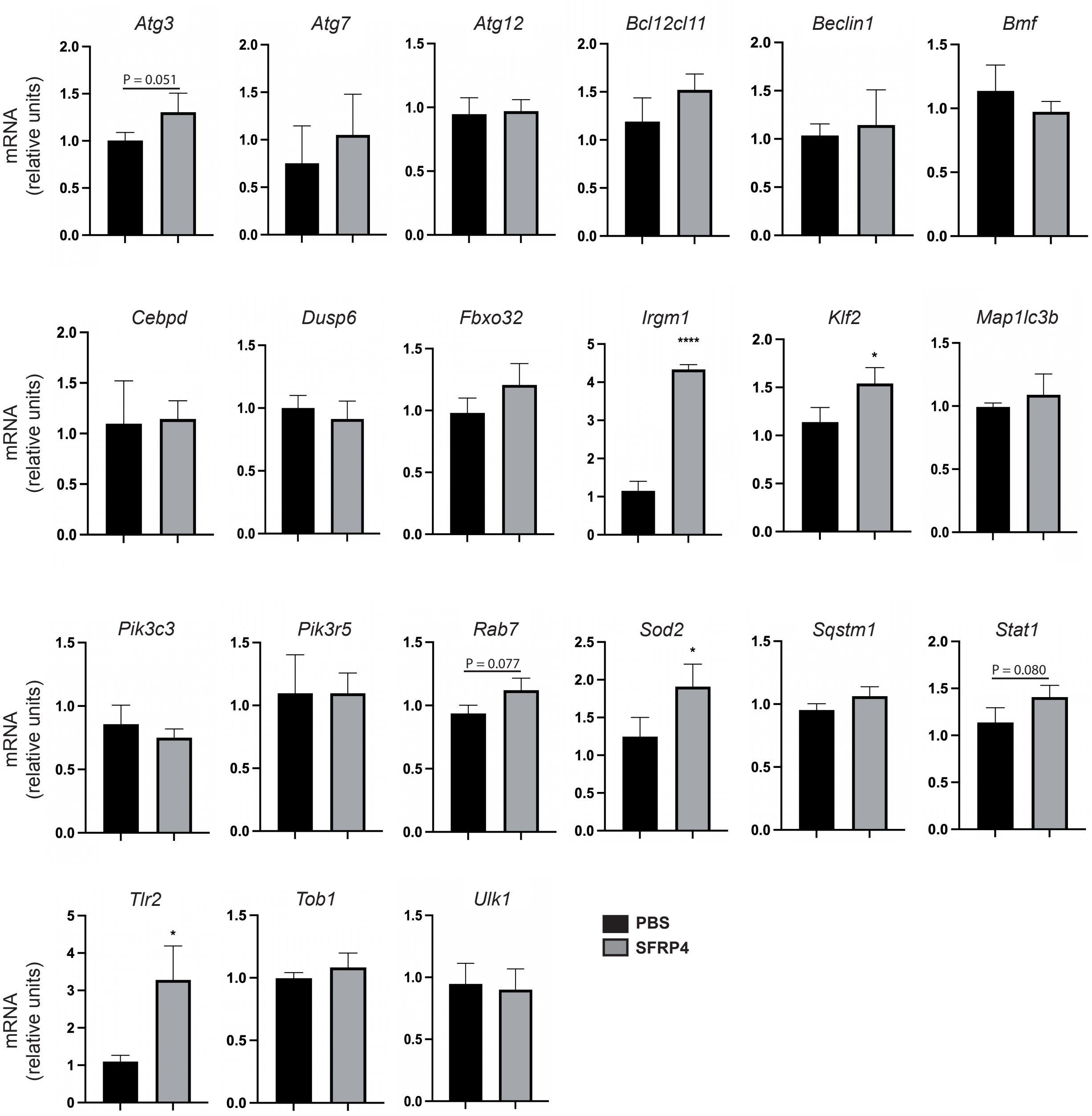

Supp. Figure1. SFRP4 regulates FOXO1-dependent autophagy-related gene expression. GCs were isolated from immature (21-26 days-old) eCG-primed wild-type mice, and placed in culture without or with recombinant SFRP4 protein (20 µg/ml) for 3h (n = 4 replicates/treatment). Data were normalized to the housekeeping gene *Rpl19*. * : $P < 0.05$; **** : $P < 0.0001$.
